# Are acute sitting-induced changes in inflammation and cerebrovascular function related to impaired mood and cognition?

**DOI:** 10.1007/s11332-021-00753-8

**Published:** 2021-04-19

**Authors:** Sophie E. Carter, Richard Draijer, Claire E. Stewart, Andy D. Moss, Dick H. J. Thijssen, Nicola D. Hopkins

**Affiliations:** 1 Research Institute for Sport and Exercise Sciences, Liverpool John Moores University, Liverpool, UK; 2 School of Science, Technology and Health, York St John University, Nestlé Rowntree Park Sports Campus, Haxby Road, York, YO31 8TA UK; 3 Unilever Foods Innovation Centre, Wageningen, The Netherlands; 4 Radboud Institute for Health Sciences, Department of Physiology, Radboud University Medical Center, Nijmegen, The Netherlands

**Keywords:** Sedentary behaviour, Sitting, Cerebral blood flow, Cerebral autoregulation

## Abstract

**Purpose:**

Sedentary behaviour is negatively associated with mood and cognition, yet how acute sitting contributes to these overall associations is unknown. Since sitting heightens inflammation and impairs cerebrovascular function, this study investigated the hypothesis that these sitting-induced changes are related to impaired mood and cognition.

**Methods:**

Twenty-five healthy desk workers (18 male, 28.3 ± 7.5 years, BMI: 24.2 ± 3.3 kg∙m^−2^) were recruited. During laboratory visit one, participants were familiarised with cognitive performance tests measuring executive function, attention and working memory. During laboratory visit two, participants completed 6 h of continuous, uninterrupted sitting. At baseline and after 6 h, serum markers of inflammation, middle cerebral artery blood flow velocity (MCAv), cerebrovascular carbon dioxide reactivity (CVR), dynamic cerebral autoregulation (CA), cognitive performance and mood (positive and negative affect, alert, contented and calm) were assessed. Data were analysed using paired-samples t tests and correlation analyses.

**Results:**

Following sitting, C-reactive protein (∆-1.0 µg/ml) and tissue plasminogen activator (∆-360.4 pg/ml) decreased (*p* < 0.05), MCAv reduced (∆-2.9 cm∙s^−1^, *p* = 0.012) and normalised gain increased in the very low frequency range, indicating impaired CA (∆ + 0.22%·mmHg^−1^, *p* = 0.016). Positive affect (∆-4.6, *p* < 0.001), and alert (∆-10.6 *p* = 0.002) and contented (∆-7.4, *p* = 0.006) mood states also decreased following sitting. No significant changes in interleukin-6, tumour necrosis factor-alpha, von Willebrand factor, CVR or cognitive performance were observed (*p* > 0.05). The observed changes in inflammation and cerebrovascular function were not related to changes in mood (*p* > 0.05).

**Conclusion:**

Alterations in inflammation or cerebrovascular function following six hours of prolonged, uninterrupted sitting are not related to the observed reductions in mood, indicating other mechanisms underlie the relationship between acute sitting and mood disturbances.

## Introduction

High amounts of sedentary behaviour (SB), any waking behaviour in a sitting, reclining or lying posture [1], are associated with clinical mood disorders, such as depression [2] and anxiety [3], and decreased cognition [4, 5]. Acute increase in sitting also negatively affects mood [6, 7] and cognition [8, 9]. Owing to the high contribution sitting has on total adult SB [10], understanding how acute sitting episodes contribute to overall associations between SB and mood and cognition is required. One potential mechanism may relate to increased inflammation. Inflammation is a critical mediator in the pathophysiology of mood disorders [11]. Acute elevation in inflammatory markers increases negative mood [12] and prolonged sitting acutely increases salivary interleukin(IL)-8 levels [13]. Furthermore, following two weeks of increased free-living SB, individuals with greater mood disturbance had an elevated IL-6 response to a stress test [14]. Sitting may therefore contribute to negative mood by enhancing inflammatory responses, but this has not been explored.

Impairments in cerebrovascular function, the regulatory mechanisms that maintain cerebral blood flow (CBF) [15], may also contribute to the associations between SB and cognition. Long-term impairment of cerebrovascular function is implicit in neurodegenerative diseases [16] and cognitive impairment [16]. Acute, prolonged sitting reduces CBF [17] and acute changes in CBF may influence cognition [18]. However, whether acute sitting-induced changes in cerebrovascular function are related to acute decrements in cognition is unknown. This study therefore investigated the relationship between sitting-induced alterations in inflammation and cerebrovascular function and changes in mood and cognition. We hypothesised that prolonged, uninterrupted sitting would increase inflammation and impair cerebrovascular function, and that these changes would be related to acute impairments in mood and cognition.

## Methods

### Participants

Twenty-five healthy desk workers (18 male) volunteered. Participants were screened prior to testing for exclusion criteria including: use of medication, smoker, BMI > 35 or < 18 kg∙m^−2^, use of hormone-based contraception and diagnosis of cerebrovascular, cardiovascular or metabolic disease. Written informed consent was obtained prior to inclusion.

### Study design

**Fig. 1:**
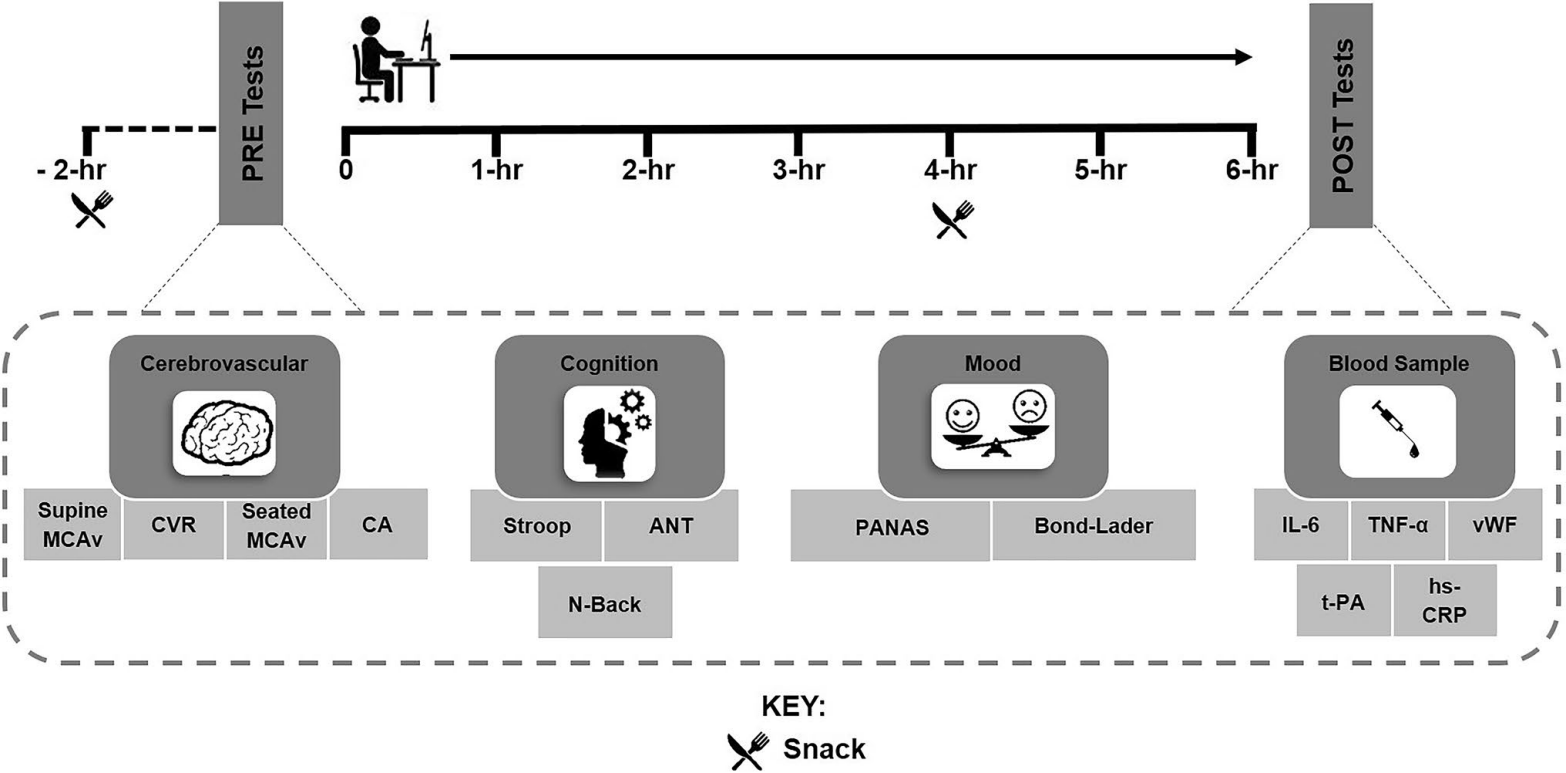
Study design for the experimental visit. *MCAv* middle cerebral artery blood flow velocity, *CVR* cerebrovascular carbon dioxide reactivity, *CA* cerebral autoregulation, *ANT* attention network test, *PANAS* positive and negative affect schedule, *IL-6* interleukin-6, *TNF-α* tumour necrosis factor-alpha, *vWF* von Willebrand factor, *t-PA* tissue plasminogen activator, *hs-CRP* high-sensitivity c-reactive protein

Study procedures were approved by the Liverpool John Moores University Ethics Committee (16/SPS/031) and conformed to the Declaration of Helsinki. Participants attended the laboratory on two occasions: (1) familiarisation visit of the cognitive performance tests; (2) experimental visit which occurred the following day. For the experimental visit, participants arrived at the laboratory between 7.00 and 9.00 am and, following a 20-min supine rest, baseline supine middle cerebral artery blood flow velocity (MCAv) and cerebrovascular carbon dioxide reactivity (CVR) were assessed. Participants were then seated and underwent measures of seated MCAv and cerebral autoregulation (CA), and a venous blood sample was collected. Following this, the same cognitive performance tests as in the familiarisation visit were undertaken and two mood questionnaires completed. After these tests (PRE), participants completed a 6 h continuous, uninterrupted sitting period. This sitting duration was based on previous research which observed reduced mood following 6 h of uninterrupted sitting [6]. The measurement of seated MCAv was repeated immediately after the 6 h intervention. MCAv was assessed while seated to examine the posture of interest, sitting, and to prevent the possible confounding haemodynamic effects of moving to a supine posture. Participants then returned to a supine posture and all other measurements were repeated (POST) (Fig. 1).

### Study procedures

#### Visit 1: familiarisation visit

Participants completed the battery of cognitive performance tests that would be used during the experimental visit to reduce any learning effects. Participants were given the opportunity to ask questions to ensure full comprehension. In preparation for the experimental visit the following day, participants were provided a standardised breakfast meal (50 g of porridge oats prepared with water and a banana) to take away with them and were instructed to consume this meal 2 h prior to their scheduled arrival time, on the morning of their second visit (Fig. 1).

#### Visit 2: experimental visit

Prior to the experimental visit, participants were instructed to avoid strenuous exercise for 24 h, and to abstain from caffeine and alcohol. Women were assessed in the follicular phase of the menstrual cycle (days 1–7) to control for hormonal variations influencing cerebrovascular responses [19]. In the 2 h between participants consuming the standardised meal and arriving at the laboratory, participants were asked to keep physical activity to a minimum aside from walking to the laboratory. On arrival, participants completed the Workforce Sitting Questionnaire (WSQ) to assess sitting time on a working day and a non-working day [20]. Participants were asked to verbally confirm they had consumed the standardised meal prior to arrival and the time at which this occurred. Participants were given this same meal 2 h before POST-measurements were taken, ensuring the time between food consumption and physiological measurements were matched between PRE- and POST-assessments (Fig. 1). Water was available to drink ad libitum. During the 6 h uninterrupted sitting period, participants remained seated at a desk and were permitted to perform low cognitively demanding desk-based activities, such as reading and watching television. Participants were prevented from standing, walking or making vigorous movements during this period, limb movements were otherwise uncontrolled (i.e. fidgeting was permitted). Participants were wheeled to the bathroom if needed (on average participants visited the bathroom once). Participants were continuously supervised to ensure these conditions were adhered to.

### Measurements

#### Blood sampling

Blood samples were obtained from the antecubital vein of the forearm via standard venepuncture technique (Vacutainers Systems, Becton–Dickinson). Samples were collected into vacutainers containing Silica (Clot Activator) and stored on ice until centrifugation for 10-min at 1,200 g at 4 °C. Serum aliquots were stored at − 80 °C for subsequent analyses. Commercially available pre-coated high-sensitivity ELISA kits (Thermo Fisher Scientific) were used to determine interleukin-6 (IL-6), tumour necrosis factor-alpha (TNF-α) and high-sensitivity c-reactive protein (hs-CRP), while commercially available pre-coated standard ELISA kits (Thermo Fisher Scientific) were used to determine tissue plasminogen activator (t-PA) and von Willebrand factor (vWF). IL-6, TNF-α and hs-CRP were analysed as they are implicated in mood disorders [21] and cognitive functioning [22]. t-PA and vWF were analysed owing to their association with reductions in CBF [23]. Kits were stored and utilised according to the manufacturer’s instructions. An automated plate reader (CLARIOstar, BMG LABTECH GmbH, Offenburg, Germany) was used to read the raw absorbance values at the 450 nm wavelength for all assays. Each sample was analysed in duplicate.

#### Middle cerebral artery blood flow velocity (MCAv)

MCAv was used as a surrogate measure for CBF as the MCA accounts for 70–80% of the brain’s total perfusion [24]. MCAv was assessed using continuous bilateral transcranial Doppler ultrasound (TCD) (ST3, Spencer Technologies, Redmond, WA, USA), with 2-MHz Doppler probes positioned over each temporal window, located above the zygomatic arch and secured using an adjustable headband (Marc 600 Headframe, Spencer Technologies). Each MCA was identified based on the signal depth, peak and mean blood velocity as previously described [15]. Once optimal signals had been obtained, the transducers were secured into position to allow measures to be collected. Probes were removed between PRE- and POST-measures. Signal parameters and photographs of the probe positions were recorded to ensure within-subject consistency between measurements. The sonographer had a between-day coefficient of variation of 7.8% for MCAv. Supine and seated MCAv were acquired for a 5-min period and mean MCAv calculated from the envelope of the velocity tracing using a weighted mean (1/3 maximum + 2/3 minimum) to account for the relative time spent in systolic and diastolic pressures [24]. Cerebrovascular conductance (CVC) was calculated by dividing MCAv by mean arterial pressure (MAP).

#### Cerebrovascular carbon dioxide reactivity (CVR)

Maintenance of adequate CBF is influenced by the brain’s ability to alter blood flow in response to changes in partial pressure of arterial carbon dioxide (CO_2_), termed CVR [25]. Testing procedures have been described in detail elsewhere [17], but briefly, participants were in a supine position and instrumented with a mouth piece (MLA1026, ADInstruments, Colorado Springs, Colorado, USA) with a two-way non-rebreathing valve (MLA1028). After a 1-min baseline, participants voluntarily hyperventilated until the pressure of end tidal CO_2_ (PETCO_2_) was reduced to 20 mmHg. Participants then returned their respiratory rate to normal and inhaled a 5% CO_2_ mixture for 3-min. MCAv was continually assessed. Simultaneously, to assess extracranial artery reactivity, left common carotid artery (CCA) arterial diameter and blood flow were measured using a 10-MHz multi-frequency linear array probe, attached to high resolution ultrasound machine (uSmart 3300; Terason, Burlington, MA, USA). Absolute and relative MCAv, CCA diameter and CCA blood flow reactivity to the changes in CO_2_ were calculated as previously described [17, 24].

#### Cerebral autoregulation (CA)

CA maintains CBF over a range of perfusion pressures [15]. To assess dynamic CA, participants completed a squat-stand test, involving repeated cycles of 5-s standing and 5-s squatting for 5-min to induce oscillations in blood pressure (BP). MCAv and BP were continuously assessed. Data were processed and analysed using standardised transfer function analysis (TFA) guidelines to produce values of gain, normalised gain, phase and coherence for each of the three frequency domains: very low frequency (VLF: 0.02–0.07 Hz), low frequency (LF: 0.07–0.2 Hz) and high frequency (HF: 0.2–0.5 Hz) [26]. These parameters are described in detail elsewhere [17].

#### Hemodynamics

Participants were fitted with a photoplethysmographic cuff on the index or middle finger of the right hand (Finometer model 1, Finapres Medical Systems BV, Amsterdam, The Netherlands) and a 3-lead electrocardiogram to continuously assess MAP and heart rate (HR), respectively, throughout measurements.

#### Mood

Mood was assessed using two questionnaires: The Positive and Negative Affect Schedule (PANAS) [27] and the Bond-Lader Mood Rating Scale [28]. The PANAS required participants to rate on a scale of 1–5 the extent to which they felt 10 positive and 10 negative states, and is a reliable measure of affect for moment in time assessments [27]. The Bond-Lader Mood Rating Scale included 12 visual analogue scales featuring bipolar end-points for different mood dimensions. These scales were combined to form three mood factors: alert, calm and contented [28].

#### Cognition

A battery of computer-based cognitive performance tests was completed using E-Prime software (Version 2.0 Professional, Psychology Software Tools, Pittsburgh, PA). Tests were completed in a randomised order between participants but not within an experimental visit. Three cognitive components were assessed: (1) *Executive function* using the Stroop Colour-Word test [29] which generated an interference score based on the reaction times from congruent and incongruent stimuli; (2) *Attention* using the Attention Network Test (ANT) which examined three attentional networks: alerting, orientating and executive control [30]; (3) *Working memory* using the N-Back Task [31] which calculated the response accuracy and time taken to identify whether a presented letter was the same as that presented one, two or three times prior in a letter sequence.

### Statistical analyses

Data were analysed using statistical software (SPSS Version 23.0, IBM Corporation, Somers, NY, USA), with significance accepted as *p* < 0.05. Results are presented as means ± standard deviation (SD). Data were assessed for normal distribution using Shapiro–Wilk tests. Differences between PRE- and POST-data were compared using paired-samples *t* tests (parametric data) or Wilcoxon signed ranked tests (non-parametric data), with post hoc analyses performed using the least significant difference (LSD) method. Effect size (Cohen’s *d*) of all significant differences was calculated by dividing the difference in group means by the standard deviation of the pooled data. These were interpreted as: *d* = 0.2 considered small, *d* = 0.5 considered medium, and *d* = 0.8 considered large [32]. Where significant changes were observed in our data following sitting, Pearson’s bivariate correlation analysis (parametric data) or Spearman’s correlation (non-parametric data) were used to assess the relationship between the changes (POST − PRE) in these outcomes.

## Results

All 25 participants completed the study and were included in analyses. Participants self-reported sitting for 12.1 ± 3.3 h during workdays and 10.0 ± 3.3 h during non-workdays. Full descriptive characteristics are shown in Table 1.

**Table 1:** Descriptive characteristics and self-reported sitting time of participants (*n* = 25)

	Mean ± SD or *n* of group
Age (years)	28.3 ± 7.5
Body Mass (kg)	74.3 ± 12.4
Height (cm)	175.0 ± 7.0
Body mass index (kg·m^−2^)	24.2 ± 3.3
Sitting time per workday (Hours)	12.1 ± 3.3
Sitting time per non-workday (Hours)	10.0 ± 3.3
Job category	
Manager/Director	1
Clerical/Services/Sales	3
Research/Technical	21

### Inflammation

Following sitting, significant decreases in t-PA (PRE: 3288.3 ± 2519.5 pg/ml, POST: 2927.9 ± 2153.8 pg/ml, *p* = 0.039, *d* = 0.16) and hs-CRP (PRE: 1.1 ± 1.3 µg/ml, POST: 0.1 ± 1.1 µg/ml, *p* = 0.028, *d* = 0.85) were observed. There were no significant changes in IL-6 (PRE: 1.9 ± 2.1 pg/ml, POST: 1.5 ± 1.7 pg/ml, *p* = 0.170), TNF-α (PRE: 1.3 ± 2.0 pg/ml, POST: 0.6 ± 1.0 pg/ml, *p* = 0.107), or vWF (PRE: 6571.6 ± 5227.2 ng/ml, POST: 6173.0 ± 3842.6 ng/ml, *p* = 0.313).

### Cardiorespiratory and haemodynamic measures

HR in the supine (*p* = 0.022, *d* = 0.48) and seated (*p* = 0.003, *d* = 0.49) postures were significantly reduced at POST compared to PRE (Table 2). There was also a significant reduction in seated MAP (*p* = 0.001, *d* = 0.56) between PRE and POST, but not for supine MAP (*p* = 0.966; Table 2). There was no significant difference between PRE and POST supine (*p* = 0.365) or seated (*p* = 0.306) PETCO_2_ (Table 2).

**Table 2:** Cardiorespiratory measures prior to (PRE) and following (POST) 6 h of uninterrupted sitting (mean ± SD)

	PRE	POST	*p*-value
Supine position			
MAP (mmHg)	84 ± 8	84 ± 8	0.966
HR (bpm)	62 ± 12	57 ± 9*	0.022
PETCO_2_ (mmHg)	38.4 ± 2.9	38.8 ± 3.6	0.365
Seated position			
MAP (mmHg)	90 ± 10	85 ± 8*	0.001
HR (bpm)	66 ± 10	61 ± 11*	0.003
PETCO_2_ (mmHg)	36.6 ± 3.0	36.8 ± 3.9	0.306

*MAP* mean arterial pressure, *HR* heart rate, *PETCO*_*2*_ pressure of end-tidal carbon dioxide

*Significantly different to PRE (*p* < 0.05)

### Middle cerebral artery blood flow velocity

**Fig. 2:**
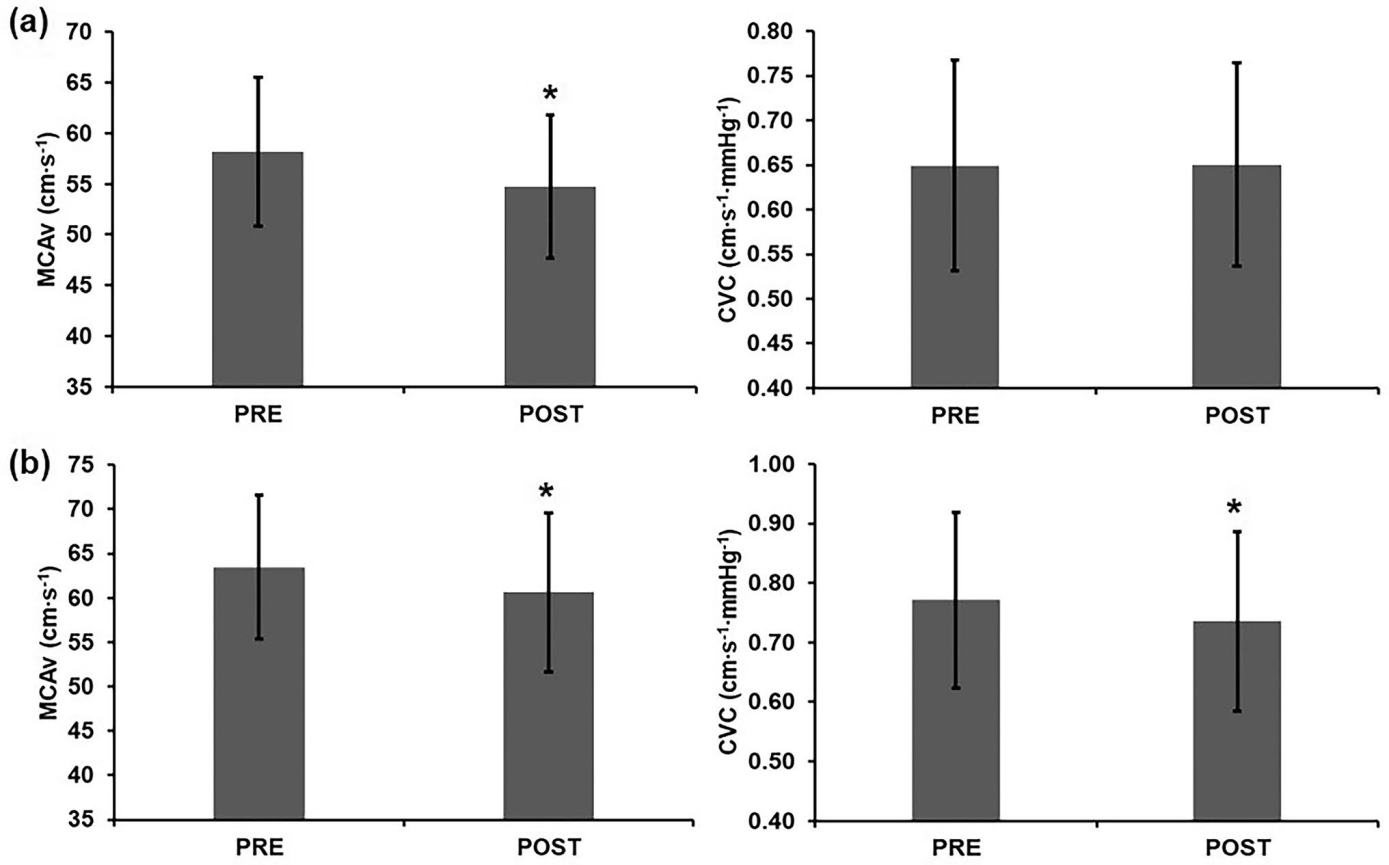
Middle cerebral artery blood flow velocity (MCAv) and cerebrovascular conductance (CVC) in **a** seated and **b** supine postures prior to (PRE) and following (POST) 6 h of uninterrupted sitting. Error bars = ± SD. *Significantly different to PRE (*p* < 0.05)

Seated MCAv significantly reduced following sitting (PRE: 58.2 ± 7.3 cm·s^−1^, POST: 54.8 ± 7.1 cm·s^−1^, *p* = 0.001, *d* = 0.48); however, there was no significant change in seated CVC (PRE: 0.65 ± 0.12 cm·s^−1^·mmHg^−1^, POST: 0.65 ± 0.11 cm·s^−1^·mmHg^−1^, *p* = 0.950; Fig. 2a). In the supine posture, significant reductions in MCAv (PRE: 63.5 ± 8.1 cm·s^−1^, POST: 60.6 ± 9.0 cm·s^−1^, *p* = 0.012, *d* = 0.35) and CVC (PRE: 0.77 ± 0.15 cm·s^−1^·mmHg^−1^, POST: 0.74 ± 0.15 cm·s^−1^·mmHg^−1^, *p* = 0.018, *d* = 0.15) were observed (Fig. 2b).

### Cerebrovascular carbon dioxide reactivity

No significant differences were observed between absolute or relative MCA CVR or CCA CVR (*p* > 0.05; Table 3).

**Table 3:** Values of cerebral autoregulation (CA) and cerebrovascular carbon dioxide reactivity (CVR) before (PRE) and after (POST) 6 h of uninterrupted sitting (mean ± SD)

	PRE	POST	*p*-value
MCA CVR			
CVR (R^2^)	0.84 ± 0.08	0.84 ± 0.09	–
Absolute MCAv (cm·s^−1^·mmHg^−1^)	3.12 ± 0.65	3.12 ± 0.79	0.992
Relative MCAv (%·mmHg^−1^)	4.82 ± 0.86	4.13 ± 1.23	0.198
CCA CVR			
Absolute CCA Diameter (cm·mmHg^−1^)	0.002 ± 0.002	0.003 ± 0.003	0.235
Relative CCA Diameter (%·mmHg^−1^)	0.33 ± 0.28	0.39 ± 0.38	0.904
Absolute CCA Blood Flow (ml·min^−1^·mmHg^−1^)	0.32 ± 0.31	0.41 ± 0.25	0.164
Relative CCA Blood Flow (%·mmHg^−1^)	2.42 ± 2.17	3.18 ± 1.99	0.122
CA: VLF			
Phase (degrees)	41.0 ± 16.7	42.1 ± 13.9	0.857
Gain (cm·s^−1^·mmHg^−1^)	0.50 ± 0.13	0.65 ± 0.17	0.051
Gain_n_ (%·mmHg^−1^)	0.86 ± 0.20	1.09 ± 0.29*	0.016
Coherence	0.5 ± 0.08	0.5 ± 0.11	–
CA: LF			
Phase (degrees)	24.4 ± 18.5	23.0 ± 13.3	0.759
Gain (cm·s^−1^·mmHg^−1^)	0.79 ± 0.18	0.85 ± 0.23	0.282
Gain_n_ (%·mmHg^−1^)	1.28 ± 0.25	1.39 ± 0.33	0.153
Coherence^#^	0.6 ± 0.11	0.6 ± 0.11	–
CA: HF			
Phase (degrees)	10.6 ± 34.8	12.4 ± 21.2	0.785
Gain (cm·s^−1^·mmHg^−1^)	0.83 ± 0.34	0.80 ± 0.27	0.727
Gain_n_ (%·mmHg^−1^)	1.34 ± 0.51	1.32 ± 0.39	0.875
Coherence^#^	0.4 ± 0.12	0.4 ± 0.13	–

*MCA* middle cerebral artery, *CVR* cerebrovascular carbon dioxide reactivity, *MCAv* middle cerebral artery blood flow velocity, *CCA* common carotid artery, *CA* cerebral autoregulation, *VLF* very low frequency, *LF* low frequency, *HF* high frequency, *Gain*_*n*_ normalised gain

^#^Coherence values were used to accept the validity of gain and phase estimates and not statistically compared

*Significantly different to PRE (*p* < 0.05)

### Cerebral autoregulation

In the VLF, there was a significant increase in normalised gain following uninterrupted sitting (*p* = 0.016, *d* = 0.94; Table 3). There were no significant changes for any other parameters in any of the frequency domains (*p* > 0.05).

### Mood

There were significant decreases in positive affect (p < 0.001, *d* = 0.62), and the alert (*p* = 0.002, *d* = 0.64) and contented (*p* = 0.006, *d* = 0.52) mood states (Table 4). Negative affect (*p* = 0.610) and the calm mood state (*p* = 0.392) did not significantly change.

**Table 4:** Mood and cognitive performance outcomes prior to (PRE) and after (POST) 6 h of uninterrupted sitting (mean ± SD)

	PRE	POST	*p*-value
Mood			
Positive affect	27.1 ± 7.2	22.5 ± 7.9*	< 0.001
Negative affect	12.5 ± 3.3	12.1 ± 2.3	0.610
Alert	53.6 ± 15.0	43.0 ± 18.5*	0.002
Calm	48.2 ± 10.6	45.9 ± 8.9	0.392
Content	67.7 ± 13.7	60.3 ± 15.5*	0.006
Executive function (Stroop Colour-Word Test)			
Interference score (ms)	185 ± 120	171 ± 133	0.425
Attention (Attention Network Test)			
Alerting network (ms)	13 ± 21	15 ± 17	0.638
Orientating network (ms)	15 ± 28	18 ± 18	0.584
Executive control (ms)	73 ± 22	75 ± 24	0.647
Working memory (N-Back Task)			
One back accuracy (%)	93.8 ± 8.6	93.0 ± 6.0	0.192
One back RT (ms)	599 ± 121	605 ± 169	0.737
Two back Accuracy (%)	92.8 ± 10.3	85.6 ± 21.3	0.153
Two back RT (ms)	877 ± 349	837 ± 294	0.437
Three back accuracy (%)	80.8 ± 17.2	75.8 ± 20.7	0.112
Three back RT (ms)	1326 ± 811	1377 ± 987	0.586

*RT* reaction time

*Significantly different to PRE (*p* < 0.05)

### Cognition

Following uninterrupted sitting, there were no significant changes in any measures of cognition (*p* > 0.05; Table 4).

### Relationship between changes in inflammation, cerebrovascular function and mood

There were no significant relationships between the changes in t-PA and hs-CRP and the changes in mood (*p* > 0.05). The changes in seated or supine MCAv and VLF normalised gain were also not significantly associated with the changes in mood (*p* > 0.05).

## Discussion

This study explored whether sitting-induced alterations in inflammation and cerebrovascular function are related to changes in mood and cognition. We observed that sitting acutely decreased levels of hs-CRP and t-PA inflammatory markers, reduced MCAv, impaired dynamic CA and decreased aspects of mood; but had no influence on cognition. The observed changes in inflammation and cerebrovascular function were not related to the changes in mood. Overall, our results indicate that neither inflammation nor cerebrovascular function are associated with this lowered mood state, suggesting other mechanisms may underlie the changes in mood in response to acute sitting.

Markers of inflammation decreased following an acute, prolonged sitting period, in contrast to our hypothesis and previous research observing increases in salivary IL-8 following an acute sitting bout [13]. Methodological differences between salivary and venous measures of inflammation could explain this disparity [33, 34]. Additionally, the controlled testing environment and completion of low cognitively demanding activities while seated may have removed participants from the daily stresses of their typical life, causing inflammatory markers to decrease. In support, acute stress activates peripheral inflammatory pathways [35], thus the removal of such stressors could have the opposite effect. This raises important methodological consideration for future experimental work assessing acute sitting and mood.

In support of previous research [17], acute sitting impaired MCAv and dynamic CA, aspects of cerebrovascular function. Importantly, the decline in MCAv (marker of CBF) following sitting is unlikely due to daily circadian variation of CBF. CBF closely tracks the rhythm of core body temperature and is therefore lower during the morning than in the afternoon or evening [36]. Since our data shows a reduction in MCAv from baseline (am) to post test (pm), this decline most likely relates to prolonged sitting rather than a circadian rhythm. Although the mechanisms of CA are not fully elucidated, it is suggested that sympathetic activity, endothelial nitric oxide production and myogenic factors all contribute [37]. In peripheral vessels, sitting-induced impairment in vascular function is likely in part due to reduced nitric oxide production and heightened sympathetic activity [38]. Similar mechanisms may therefore contribute to the impaired CA observed in this study.

Prolonged sitting negatively impacted aspects of mood, supporting previous observations that reducing sitting acutely improves mood [6, 7]. Heightened inflammation may contribute to sitting-induced decreases in mood [14]; however, inflammation decreased in our study. Consequently, it was unsurprising that the changes in mood were not associated with any inflammatory markers as there would be no underlying physiological reason for a relationship between these variables. Furthermore, the decreases in mood were not correlated with the reductions in MCAv or CA we observed, indicating cerebrovascular function may not be a mechanism explaining acute mood alterations. An alternative mechanism relating to acute, prolonged sitting may therefore explain our mood data. Indeed, the reduction in mood may relate to the laboratory testing setting and participant boredom, which cannot be ruled out as potential contributing factors.

No changes in cognition were observed following sitting, which contrasts previous research in older adults [9] and Qatari females [8]. Differences between our findings may relate to the variety of tests used to assess each cognitive domain, in addition to the populations assessed, since other studies have observed no influence on cognition following acute sitting bouts when assessing younger, adult males and females [6, 39]. It has been hypothesised that hypoperfusion of the brain due to prolonged sitting contributes to cognitive decline [40], but despite decreases in MCAv observed in this study, this did not translate to changes in cognition, indicating that in an acute setting changes in MCAv are not associated with cognition. However, MCAv does not measure regional blood flow and it has been recently observed that prefrontal cortex perfusion was unaltered following an acute sitting bout and executive function was also unchanged [41]. This suggests that acutely, whilst MCAv decreases, perfusion and oxygen delivery to specific brain regions is maintained, which may preserve cognition. Instead, chronic exposure to sitting-induced decreases in MCAv may cause structural and function damage, leading to long-term cognitive impairment. Future research should explore these mechanisms in more detail.

### Limitations

The cognitive tests selected may not have been sensitive enough to detect an effect of sitting, or we may have assessed domains of cognition that are not influenced by sitting. A learning effect may have also occurred for the cognitive tests, although the inclusion of a familiarisation visit aimed to reduce this risk. The desk-based activities participants completed during sitting were not controlled, therefore they may have differentially influenced cerebrovascular, cognitive and mood responses. Due to the length of the experimental protocol, measurements could not be completed in a fasted state as is usual best practice. However, the timing and content of the meals prior to each measurement time-point were matched, so any postprandial influence on outcomes measures would be similar.

## Conclusion

This study demonstrates that in healthy desk workers, alterations in inflammation or cerebrovascular function following 6 h of prolonged, uninterrupted sitting are not related to the observed reductions in mood. Overall, this study provides initial, exploratory data that future experimental research should investigate further to determine the mechanisms underlying the influence of sitting on mood and cognition.

## Data Availability

The datasets used and/or analysed during the current study are available from the corresponding author on reasonable request.

## Abbreviations

BP: Blood pressure
CO_2_: Carbon dioxide
CA: Cerebral autoregulation
CBF: Cerebral blood flow
CVR: Cerebrovascular carbon dioxide reactivity
CVC: Cerebrovascular conductance
CCA: Common carotid artery
HR: Heart rate
HF: High frequency
hs-CRP: High-sensitivity c-reactive protein
IL-6: Interleukin-6
LF: Low frequency
MAP: Mean arterial pressure
MCAv: Middle cerebral artery blood flow velocity
PANAS: Positive and negative affect schedule
PETCO_2_: Pressure of end tidal CO_2_
SB: Sedentary behaviour
t-PA: Tissue plasminogen activator
TNF-α: Tumour necrosis factor-alpha
VLF: Very low frequency
vWv: von Willebrand factor
